# Risk-stratified multi-round PSA screening for prostate cancer integrating the screening reference level and subgroup-specific progression indicators

**DOI:** 10.1186/s40001-023-01228-x

**Published:** 2023-07-26

**Authors:** Xiaomin Liu, Yu Zhang, Hongyuan Duan, Lei Yang, Chao Sheng, Zeyu Fan, Ya Liu, Ying Gao, Xing Wang, Qing Zhang, Zhangyan Lyu, Fangfang Song, Fengju Song, Yubei Huang

**Affiliations:** 1 Department of Epidemiology and Biostatistics, Key Laboratory of Molecular Cancer Epidemiology (Tianjin), National Clinical Research Center for Cancer, Tianjin’s Clinical Research Center for Cancer, Tianjin Medical University Cancer Institute and Hospital, Tianjin Medical University, Hexi District, Tianjin, 300060 China; 2 Key Laboratory of Carcinogenesis and Translational Research (Ministry of Education), Beijing Office for Cancer Prevention and Control, Peking University Cancer Hospital & Institute, Beijing, 100142 China; 3 Health Management Center, Tianjin Medical University General Hospital, Tianjin, 300060 People’s Republic of China

**Keywords:** Prostate cancer, PSA, Screening, Progress, Velocity

## Abstract

**Background:**

Although prostate-specific antigen (PSA) is widely used in prostate cancer (PCa) screening, nearly half of PCa cases are missed and less than one-third of cases are non-lethal. Adopting diagnostic criteria in population-based screening and ignoring PSA progression are presumed leading causes.

**Methods:**

A total of 31,942 participants with multi-round PSA tests from the PLCO trial were included. Time-dependent receiver-operating-characteristic curves and area under curves (tdAUCs) were performed to determine the screening reference level and the optimal subgroup-specific progression indicator. Effects of risk-stratified multi-round PSA screening were evaluated with multivariable Cox regression and measured with hazard ratio [HR (95%CIs)].

**Results:**

After a median follow-up of 11.6 years, a total of 3484 PCa cases and 216 PCa deaths were documented. The tdAUC of 10-year incidence PCa with PSA was 0.816, and the cut-off value was 1.61 ng/ml. Compared to subgroup with stable negative PSA in both first-round (FR) and last-round (LR) tests [FR(−)/LR(−)], HRs (95%CI) of PCa incidence were 1.66 (1.20–2.29), 8.29 (7.25–9.48), and 14.52 (12.95–16.28) for subgroups with loss of positive PSA[FR(+)/LR(−)], gain of positive PSA[FR(−)/LR(+)], and stable positive PSA[FR(+)/LR(+)]; while HRs(95%CI) of PCa mortality were 1.47 (0.52–4.15), 5.71 (3.68–8.86), and 5.01 (3.41–7.37). After excluding regressive PSA [(namely FR(+)/LR(−)], absolute velocity was the shared optimal progression indicator for subgroups with FR(−)/LR(−), FR(−)/LR(+), and FR(+)/LR(+), with tdAUCs of 0.665, 0.681 and 0.741, and cut-off values of 0.07, 0.21, and 0.33 ng/ml/year. After reclassifying participants into groups with positive and negative progression based on subgroup-specific progression indicators, incidence HR (95%CI) were 2.41 (1.87–3.10), 2.91 (2.43–3.48), and 3.16 (2.88–3.46) for positive progression compared to negative progression within subgroups of FR(−)/LR(−), FR(−)/LR(+), and FR(+)/LR(+), while mortality HR (95%CI) were 2.22 (0.91–5.38), 2.37 (1.28–4.38), and 2.98 (1.94–4.59). To improve screening performances by excluding regressive PSA and low-risk positive progression in FR(−)/LR(−), optimized screening strategy not only significantly reduce 32.4% of missed PCa (54.0% [1881/3484] vs. 21.6% [754/3484], P < 0.001), but also detected additional 8.0% of high-grade PCa (Gleason score 7–10: 36.0% [665/1849] vs. 28.0% [206/736], P < 0.001) than traditional screening strategy.

**Conclusions:**

Risk-stratified multi-round PSA screening strategy integrating the screening reference level and the optimal subgroup-specific progression indicator of PSA could be recommended as a fundamental strategy to reduce missed diagnosis and improve the detection of high-grade PCa cases.

**Supplementary Information:**

The online version contains supplementary material available at 10.1186/s40001-023-01228-x.

## Introduction

Prostate cancer (PCa) is the most common cancer and second leading cause of cancer death in men worldwide [1]. In addition to efforts to improve treatment and primary prevention of PCa, prostate-specific antigen (PSA) screening has long been recognized as effective strategy to reduce the PCa burden and has been widely used in western countries for decades [2]. As shown in the European Randomized Study of Screening for Prostate Cancer (ERSPC), PSA screening significantly reduces PCa mortality by 20% in men aged 50 to 74 years [3]. Moreover, the PROBASE study suggested that risk-adapted PSA screening can potentially inhibit PCa progression to metastatic disease, and that the prevalence of screen-detected invasive PCa was very low in 45-year-old men [4]. However, several other trials with extended follow-up do not support the benefits of PSA screening for PCa mortality, including the Cluster Randomized Trial of PSA Testing for Prostate Cancer (CAP) and the Prostate, Lung, Colorectal, and Ovarian cancer screening trial (PLCO) [5–7]. In addition to PSA contamination in the control arm and potential overdiagnosis associated with screening, potential missed PCa under traditional screening strategy and low proportion of clinically significant PCa among screening-detected PCa would be another key explanations for the lack of a reduction in mortality observed in both PLCO and CAP [5, 8–12].

Adoption of PSA diagnostic criteria for PCa (4 ng per milliliter [ml]) in population-based screening is presumed to be the leading cause of the missed PCa associated with traditional PSA screening strategy. Since most screening population are healthy men, and only a minority of men have PSA above the diagnostic criteria. Several recent studies had reported that approximately half of the tumors were missed with PSA 0 to 4 ng/ml but with aggressive characteristics (Gleason score 7 or greater) [10–14]. Therefore, it is necessary to redefine the population-based appropriate PSA screening reference level for population-based PCa screening. Moreover, the Finnish section of ERSPC indicated that at least three rounds of PSA screening were needed to reduce subsequent PCa incidence and mortality [8, 9], which indicated multi-round PSA tests are necessary to enhance the effectiveness of screening for prostate cancer.

To improve the detection of clinically significant high-grade or lethal PCa cases, previous studies have proposed several strategies to cope with the above limitations of PSA screening. As observed in STHLM3-MRI, a combination of risk prediction, magnetic resonance imaging (MRI), and targeted prostate biopsies has shown the ability to detect clinically significant cancer [15, 16]. However, MRI is not readily available in many resource-limited settings or even well-resource regions. Additionally, several PSA progression indicators based on repeated PSA tests have been also proposed to improve the effectiveness of PSA screening, such as PSA velocity and doubling time, etc [17–21]. However, most of these progression indicators are used for prognostic monitoring of PCa, and few studies have investigated and compared the performance of different PSA progression indicators under the setting of population-based screening.

In summary, based on the PLCO trial, the main purpose of this study is to determine the PSA reference level for population-based PCa screening and then to determine the optimal subgroup-specific progression indicators and their subgroup-specific cut-off values under the setting of population-based screening. After integrating the above indicators, we finally aim to propose an optimized risk-stratified multi-round PSA screening strategy to reduce the missed PCa associated with traditional screening strategy and improve the detection of clinically significant PCa.

## Materials and methods

### Source of population

Data of participants in this study were collected from the online datasets of the PLCO trial (https://cdas.cancer.gov/plco/). Cancer data collected up to December 31, 2009, and mortality data collected through 2015 for each participant in the PLCO trial are available on this website. The design of the PLCO Cancer Screening Trial has been described previously [22–24]. In brief, a total of 76,683 men aged 55 to 74 years during 1993–2001 were recruited from 10 cancer screening centers across the United States. Each institution obtained approval from its institutional review board, and all participants provided written informed consent. Participants were individually randomized to the control arm or intervention arm within blocks stratified according to center, age, and sex in equal proportions. Participants assigned to the control arm received usual care, while participants assigned to the intervention arm were invited to receive screening exams for prostate, lung, colorectal, and ovarian cancers as outlined in the study protocol. Subjects assigned to the PCa screening were offered annual PSA testing for 6 years and annual digital rectal examination (DRE) for 4 years [5, 22, 23].

In the PLCO trial, of the 37,282 eligible men who completed the baseline questionnaire after informed consent in the intervention arm, we initially excluded a total of 802 men with a history of cancer, 1112 men with prostatectomy, and 1687 men who did not receive any PSA tests. Among the remaining 33,681 participants who received PSA test, we further excluded 1672 participants who received only one PSA test, and 67 PCa patients with only one PSA test before diagnosis. Finally, a total of 31,942 participants in the analytic cohort were included in this study (Additional file 1: Fig. S1).

### Follow-up and outcome ascertainment

Men with any PSA > 4 ng/ml or any suspicious abnormality on DRE were considered to have a positive screening and they were recommended to seek diagnostic evaluation, which was decided by the patients and their primary physician [25, 26]. Staff at each PLCO study center obtained and recorded medical information related to diagnostic evaluation. PCa cases were ascertained by a combination of abovementioned diagnostic evaluation after positive screening, an annual study update (ASU) form inquired about cancer diagnoses, and periodic linkage to the National Death Index (NDI) for participants who did not responded to ASU form [22–24]. All cancer characteristics were documented according to the cancer staging manual by the American Joint Committee on Cancer (AJCC).

Deaths were ascertained primarily by the ASU form and supplemented by periodic linkage to NDI. Once any deaths were notified via the ASU form or NDI, PLCO staff obtained death certificates from state bureaus of vital statistics and collected complete mortality data coded in line with the ninth edition of International Classification of Diseases (ICD-9). The causes of death were reviewed by an end-point adjudication process for those without evident or accurately recorded underlying cause on the death certificate.

### Assessment of covariates

After informed consent, each participant was provided with a baseline risk factor questionnaire to collect participant-reported information on demographics, smoking history, family history of cancer, height, weight, non-steroidal anti-inflammatory drugs (NSAIDs), medical conditions and history of disease, sex-specific information (enlarged prostate, prostatitis, prostate surgery, etc.), and screening prior to baseline. Body mass index (BMI) was calculated as weight in kilograms divided by the square of height in meters (kg/m^2^). Demographics included age at entrance, race (white, black, other), education, marital status, occupation, etc. Medical conditions included history of enlarged prostate, prostatitis, diabetes, heart disease, stroke, hypertension and other diseases. After excluding variables that were not significantly associated with PCa in the univariate analyses, all of the following potential confounding factors were initially included in the multivariable analyses: age at entrance (< 60, 60–70, ≥ 70 years), race (white, black, other), BMI (0–25, 25–30, > 30 kg/m^2^), smoking status (never, current, former), family history of prostate cancer (no, yes), history of previous PSA screening (no, 1 time, ≥ 2 times), history of enlarged prostate (no, yes), history of diabetes (no, yes), and the results of first- and last-round DRE (Additional file 1: Table S1).

### Statistical analyses

To investigate the population-based PSA screening reference level for incidence PCa, univariate Cox regression with baseline first-round PSA level was performed. Time-dependent receiver-operating-characteristic curves (tdROCs) and area under curves (tdAUCs) were performed to document the 10 years risk of PCa and determine the risk prediction accuracy. The optimal cut-off value of tdROCs was defined as the population-based screening reference level, and the participants were reclassified into the following four subgroups based on first-round and last-round (FR and LR) PSA according to the screening reference level: stable negative PSA subgroup with negative PSA in both FR and LR PSA tests [FR(−)/LR(−)], loss of positive PSA subgroup with FR-positive PSA and LR-negative PSA [FR(+)/LR(−)], stable positive PSA subgroup with positive PSA in both FR and LR PSA tests [FR(+)/LR(+)], and gain of positive PSA subgroup with FR-negative PSA and LR-positive PSA [FR(−)/LR(+)].

Among subgroups with elevated PSA, including FR(−)/LR(−), FR(−)/LR(+), and FR(+)/LR(+), eight progression indicators were calculated: absolute increment (AbsInc) defined as the difference between FR and LR PSA level, maximum absolute increment (MaxAbsInc) defined as the difference between FR PSA and maximum PSA level during repeated PSA tests, relative increment (RelInc) defined as AbsInc divided by FR PSA level, maximum relative increment (MaxRelInc) defined as MaxAbsInc divided by FR PSA level, absolute velocity (AbsVel) defined as AbsInc divided by the duration in year between FR and LR PSA tests, maximum absolute velocity (MaxAbsVel) defined as MaxAbsInc divided by the duration in year between FR and maximum PSA tests, relative velocity (RelVel) defined as AbsVel divided by FR PSA level, and maximum relative velocity (MaxRelVel) defined as MaxAbsVel divided by FR PSA level. To determine the prediction accuracy of these PSA progression indicators for long-time incidence of PCa, tdAUCs of 10-year incidence PCa were calculated using univariate Cox regressions. The Delong test was used to compare whether any pairwise tdAUC was significantly different within specific subgroup, and the indicator with the largest tdAUC was defined as the optimal subgroup-specific progression indicator. Both the PSA screening reference level and cut-off values of subgroup-specific PSA progression indicator were internally validated using bootstrap resampling for 2000 iterations and 95% confidence intervals (CIs) were calculated.

The optimal cut-off values of subgroup-specific progression indicator were further used to reclassified the abovementioned three subgroups with elevated PSA into the following two subgroups: subgroup with negative progression [Prog (−)] defined as PSA progression less than or equal to the subgroup-specific cut-off value, and subgroup with positive progression [Prog (+)] defined as PSA progression greater than the subgroup-specific cut-off value. Log-rank test was first used to compare the differences of PCa incidence and mortality between the two subgroups. Multivariable Cox regression analyses were performed to evaluate the independent effects of subgroup-specific progression indicator on PCa incidence and mortality after adjusting all available confounding factors described above and the rounds of screening between FR and LR PSA tests. The relative risks were measured as hazard ratio and 95% confidence intervals (CIs).

Based on the screening reference level and the optimal subgroup-specific progression indicator of PSA, we proposed three optimized PSA screening strategies parallel to traditional PSA screening. For the traditional PSA screening, positive screen was defined as any PSA above diagnostic criteria (any PSA > 4 ng/ml). For the first optimized PSA screening strategy (simplified as optimized strategy one), positive screen was defined as any PSA above population-based screening reference level. To further reduce potential missed diagnosis of PCa cases, positive screen in the second optimized PSA screening strategy (simplified as optimized strategy two) included both positive screen in optimized strategy one and any positive progression in subgroups with stable negative PSA[(FR(−)/LR(−)], gain of positive PSA[(FR(−)/LR(+)], and stable positive PSA[(FR(+)/LR(+)]. To reduce potential false positive and improve the detection of high-grade PCa cases, positive screen in the third optimized PSA screening strategy (simplified as optimized strategy three) excluded low-risk positive screen in optimized strategy two, including positive screen in the subgroup with loss of positive PSA [(FR(+)/LR(−)] and positive progression in subgroup with stable negative PSA[(FR(−)/LR(−)]. Screening performances [including sensitivity, specificity, positive prediction value (PPV), negative prediction value (NPV), proportion of high-grade PCa (defined as Gleason score 7–10), and proportion of false positive (defined as the number of participants with false positive divided the total number of all participants)] for different screening strategies were further calculated and compared with Pearson Chi-square tests.

All analyses were performed with R (version 4.1.2) and SPSS (version 25.0). The survival curves were drawn by the package “survminer” (version 0.4.9) in the R software, and the tdROCs were drawn by the package “riskRegression” (version 2021.10.10). The *P* value < 0.05 (two-tailed) was considered statistically significant.

## Results

### Determination of the population-based PSA screening reference level

**Fig. 1:**
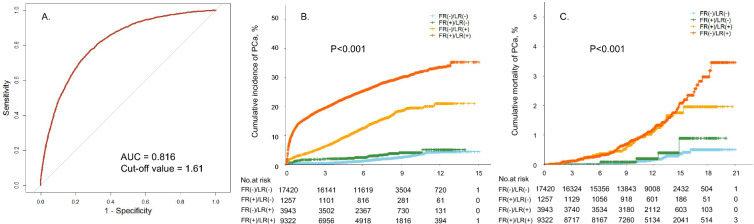
Time-dependent receiver-operating-characteristic curves of 10-year incidence of prostate cancer with PSA (**A**) and Kaplan–Meier curves of cumulative prostate cancer incidence (**B**) and mortality (**C**) for different subgroups according to population-based screening reference value. Note: FR(−)/LR(−), stable negative PSA; FR(+)/LR(−), loss of positive PSA; FR(−)/LR(+), gain of positive PSA; FR(+)/LR(+), stable positive PSA

After a median follow-up of 11.6 years, a total of 3484 PCa cases and 216 PCa deaths were documented among 31,942 participants. As shown in Fig. 1A, tdAUC of 10-year incidence PCa for FR PSA level was 0.816, and the optimal cut-off value of FR PSA level for 10-year incidence PCa was 1.61 ng/ml, which was selected as the population-based PSA screening reference level. After internal validation with bootstrap resampling analysis, the median cut-off value of screening reference level was 1.60 ng/ml (95%CI 1.47–1.86), which was similar to above preliminary analysis (Additional file 1: Table S2). Based on the screening reference level, 33.1% (10,579/31,942) and 66.9% (21,363/31,942) of participants were reclassified into two subgroups with FR positive or negative PSA. As shown in Additional file 1: Fig. S2, subgroup with FR-positive PSA showed significant higher risks of PCa incidence and mortality (both *P* values < 0.001) than FR-negative PSA, even after adjusting potential confounders (data not shown).

### Effects of multi-round PSA screening based on population-based screening reference value

After integrating FR and LR PSA and reclassifying the participants according to the screening reference level, a total of 54.5%, 3.9%, 12.3% and 29.2% of the participants were reclassified into subgroups with FR(−)/LR(−), FR(+)/LR(−), FR(−)/LR(+) and FR(+)/LR(+). The crude subgroup-specific incidences of PCa gradually increased from 2.81, 4.79, and 22.22, to 46.34 per 1000 person years, while crude PCa mortality were 0.18, 0.29, 1.03, and 1.19 per 1000 person years, respectively (Additional file 1: Table S3). Kaplan–Meier curves showed significant differences on the crude PCa incidences and mortalities between the four subgroups (Fig. 1B and C, both *P* values < 0.001). After adjusting confounding factors and compared to subgroups with FR(−)/LR(−), HRs (95%CI) of PCa incidence were 1.66 (1.20–2.29), 8.29 (7.25–9.48), and 14.52 (12.95–16.28) for subgroups with FR(+)/LR(−), FR(−)/LR(+) and FR(+)/LR(+), while HRs (95%) of PCa mortality were 1.47 (0.52–4.15), 5.71 (3.68–8.86), and 5.01 (3.41–7.37), respectively (Additional file 1: Table S3). Further interaction analyses showed similar HRs of PCa incidence and mortality for subgroups with FR(+)/LR(−), FR(−)/LR(+) and FR(+)/LR(+) compared to subgroups with FR(−)/LR(−) across different rounds of PSA screening (Additional file 1: Table S4).

### Determination of the subgroup-specific progression indicator and its optimal cut-off values

**Fig. 2:**
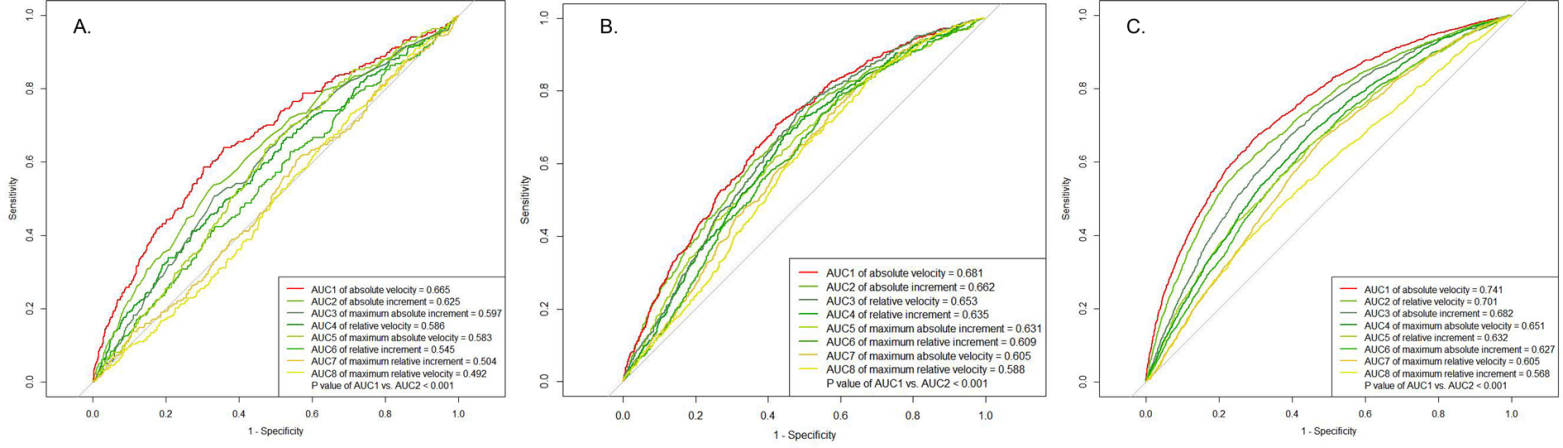
Time-dependent receiver-operating-characteristic curves of 10-year incidence of prostate cancer with different PSA progression indicators in men with stable negative PSA (**A**), gain of positive PSA (**B**) and stable positive PSA (**C**)

Figure 2 shows the tdROCs and tdAUCs of 10-year incidence PCa with eight progression indicators in three subgroups with elevated PSA, including subgroups with FR(−)/LR(−), FR(−)/LR(+) and FR(+)/LR(+). After pairwise comparison of index tdAUC with the maximum tdAUC within the same subgroups, absolute velocity (AbsVel) was identified as the shared optimal PSA progression indicator among eight indicators (all *P* values < 0.05). The tdAUCs of 10-year incidence PCa for AbsVel were 0.665, 0.681 and 0.741 for subgroups with FR(−)/LR(−), FR(−)/LR(+) and FR(+)/LR(+), respectively, while the subgroup-specific cut-off values of AbsVel were 0.07, 0.21 and 0.33 ng/ml/year, respectively. After bootstrap resampling analysis, the median cut-off values of AbsVel for the above three subgroups were 0.07 (95%CI 0.06–0.10), 0.23 (95%CI 0.20–0.28), 0.37 (95%CI 0.31–0.42), respectively (Additional file 1: Table S2).

### Effects of subgroup-specific PSA progression indicator

After further reclassifying participants into Prog(+) and Prog(−) subgroups according to optimal cut-off values of subgroup-specific progression indicator, a total of 29.1% of subgroups with FR(−)/LR(−), 54.2% of subgroups with FR(−)/LR(+), and 41.1% of subgroups with FR(+)/LR(+) were reclassified into subgroups with Prog(+) (Table 1). In above three subgroups with elevated PSA, Kaplan–Meier curves showed that the cumulative incidence and mortality of PCa in subgroup with Prog(+) was significantly higher than that of subgroup with Prog(−) (Additional file 1: Fig. S3). After further adjusting all available confounding factors and rounds of PSA screening, incidence HR(95%CI) for Prog(+) compared to Prog(−) were 2.41 (1.87–3.10), 2.91 (2.43–3.48), and 3.16 (2.88–3.46) for subgroups with FR(−)/LR(−), FR(−)/LR(+) and FR(+)/LR(+), while mortality HR (95%) were 2.22 (0.91–5.38), 2.37 (1.28–4.38), and 2.98 (1.94–4.59), respectively (Table 1).

**Table 1:** Associations of PSA progression with prostate cancer (PCa) incidence and mortality

Subgroups	PSA progression	Participants, N (%)	Event, N (%)	Follow-up, 1000 PYs	Event rate, per 1000 PYs	Adjusted HR (95%CI)^a^	*P* value^a^
PCa incidence							
FR(−)/LR(−)	Prog(−)	7766(70.9)	121(46.0)	55.06	2.20	Ref.	
	Prog(+)	3187(29.1)	142(54.0)	22.97	6.18	2.41(1.87–3.10)	< 0.001
FR(−)/LR(+)	Prog(−)	2159(54.8)	181(31.4)	14.88	12.16	Ref.	
	Prog(+)	1784(45.2)	396(68.6)	11.08	35.75	2.91(2.43–3.48)	< 0.001
FR(+)/LR(+)	Prog(−)	4172(58.9)	760(34.8)	27.23	27.91	Ref.	
	Prog(+)	2906(41.1)	1485(65.2)	12.03	123.41	3.16(2.88–3.46)	< 0.001
PCa mortality							
FR(−)/LR(−)	Prog(−)	7766(70.9)	10(47.6)	90.26	0.11	Ref.	
	Prog(+)	3187(29.1)	11(52.4)	36.83	0.30	2.22(0.91–5.38)	0.079
FR(−)/LR(+)	Prog(−)	2159(54.8)	16(34.0)	25.30	0.63	Ref.	
	Prog(+)	1784(45.2)	31(66.0)	20.45	1.52	2.37(1.28–4.38)	0.006
FR(+)/LR(+)	Prog(−)	4172(58.9)	32(26.4)	48.80	0.66	Ref.	
	Prog(+)	2906(41.1)	89(73.6)	34.52	2.58	2.98(1.94–4.59)	< 0.001

PY, person-year; HR (95%CI), hazard ratio (95% confidential interval); FR(−)/LR(−), stable negative PSA; FR(−)/LR(+), gain of positive; FR(+)/LR(+), stable positive PSA; Prog, progression; -, negative;+ , positive

^a^Adjusted all potentially confounding factors mentioned in the method

Moreover, as shown in Additional file 1: Fig. S4 and Table S5 with cumulative incidence and mortality of PCa between different subgroups integrating the screening reference level and subgroup-specific progression indicators**,** compared to FR(−)/LR(−)/Prog(−), FR(+)/LR(+)/Prog(+) showed the highest PCa incidence and mortality [adjusted HR (95%CI): 44.53 (38.30–51.77) and 12.15 (7.56–19.51)], followed by FR(−)/LR(+)/Prog(+) [16.34 (13.78–19.37) and 9.14 (5.35–15.61)], FR(+)/LR(+)/Prog(−) [11.61 (9.97–13.52) and 3.02 (1.82–5.04)], and FR(−)/LR(+)/Prog(−) [6.41 (5.24–7.84) and 4.27 (2.27–8.05)]. No significant difference was found in the mortality of PCa between FR(−)/LR(−)/Prog(−), FR(−)/LR(−)/Prog(+), and FR(+)/LR(−).

### Effects of risk-stratified multi-round PSA screening integrating population-based screening reference value and subgroup-specific progression indicator

As shown in Table 2, based on diagnostic criteria of PSA for PCa, the sensitivity, specificity, PPV and NPV were 21.6%, 96.3%, 46.2% and 90.9%, respectively. Compared to traditional PSA screening, after lowering the positive criterion of PSA to the screening reference value, the sensitivity and NPV of optimized strategy one increased to 73.6% and 95.7%, but the specificity and PPV decreased to 71.8% and 24.2%. After further including any subgroup-specific positive progression as positive screen, the sensitivity and NPV of optimized strategy two further increased to 94.2% and 98.6%, while the specificity and PPV further decreased to 49.3% and 18.5%. To reduce potential false positive and improve the detection of high-grade PCa cases after excluding low-risk positive screen in optimized strategy two, the sensitivity and NPV of optimized strategy three decreased to 54.0% and 94.1%, while the specificity and PPV increased to 90.1% and 40.1%.

**Table 2:** Comparison of screening performances between different screening strategies

Methods	Cases	Non-cases	Total	Sensitivity		Specificity		PPV		NPV	
				%	*P*	%	*P*	%	*P*	%	*P*
Traditional PSA screening (positive screen defined as any PSA > 4 ng/ml)											
Positive	754	1330	2084	21.6	Ref.	96.3	Ref.	46.2	Ref.	90.9	Ref.
Negative	2730	27,128	29,858								
Total	3484	28,458	31,942								
Optimized strategy 1 (positive screen defined as any PSA > 1.61 ng/ml)											
Positive	2564	8015	10,579	73.6	< 0.001	71.8	< 0.001	24.2	< 0.001	95.7	< 0.001
Negative	920	20,443	21,363								
Total	3484	28,458	31,942								
Optimized strategy 2 (positive screen included all positive screen in optimized strategy 1 and any subgroup-specific positive progression)^†^											
Positive	3283	14,426	17,709	94.2	< 0.001	49.3	< 0.001	18.5	< 0.001	98.6	< 0.001
Negative	201	14,032	14,233								
Total	3484	28,458	31,942								
Optimized strategy 3 (positive screen excluded low-risk positive screen in optimized strategy 2)^‡^											
Positive	1881	2809	4690	54.0	< 0.001	90.1	< 0.001	40.1	< 0.001	94.1	< 0.001
Negative	1603	25,649	27,252								
Total	3484	28,458	31,942								

PPV: †, subgroup-specific positive progression including positive progression in subgroups with stable negative PSA[(FR(−)/LR(−)], gain of positive PSA[(FR(−)/LR(+)], and stable positive PSA[(FR(+)/LR(+)]; ‡, low-risk positive screen in optimized strategy 2 included positive screen in the subgroup with loss of positive PSA [(FR(+)/LR(−)] and positive progression in subgroup with stable negative PSA[(FR(−)/LR(−)]. PPV, positive predictive value; NPV: negative predictive value

**Fig. 3:**
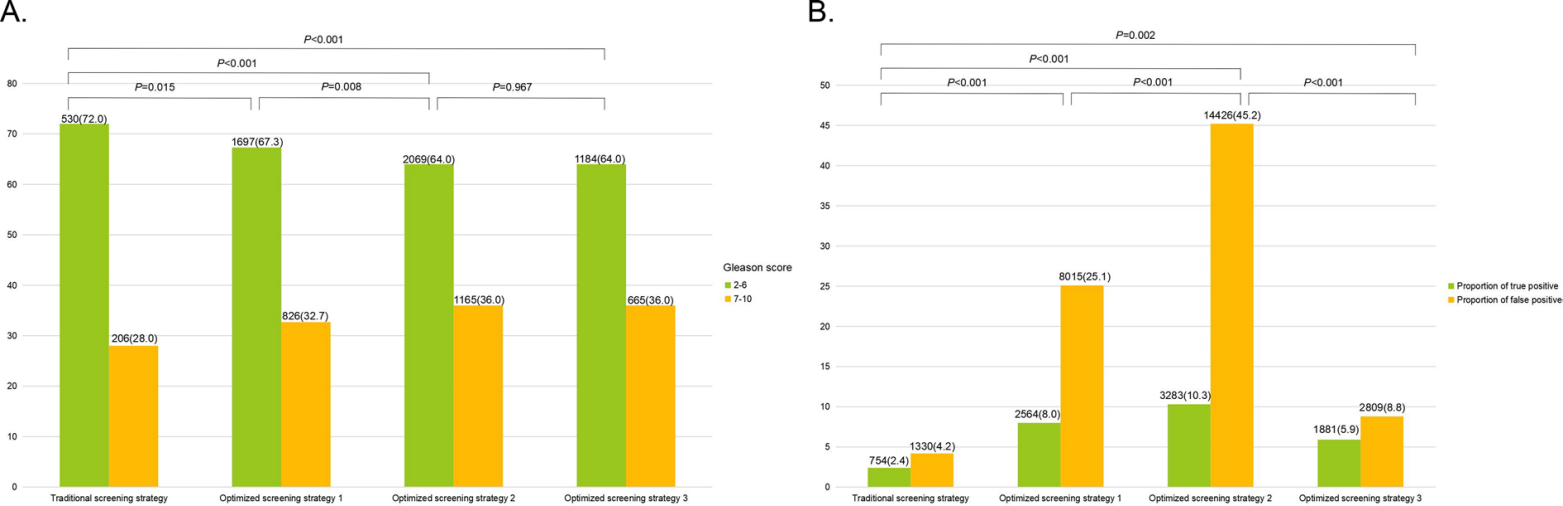
Comparison of high-grade prostate cancer (Gleason score greater than 7) (**A**) and proportions of true/false positive (**B**) between different screening strategies. Proportions of true/false positive were defined as the number of participants with true/false positive divided the total number of participants

More importantly, as shown in Fig. 3A, compared to proportion of high-grade PCa (Gleason score 7–10) detected by traditional PSA screening (28.1%), any optimized screening strategies detected significantly higher proportion of high-grade PCa (32.7%, 36.0%, and 36.0%, all *P* values < 0.05). Additionally, although there was no difference in the proportions of high-grade PCa between optimized screening strategy two and three (*P* = 0.967), the proportion of false-positive PSA in optimized strategy three was significantly lower than that in optimized strategy two (8.8% vs. 45.2%, *P* < 0.001) (Fig. 3B).

**Fig. 4:**
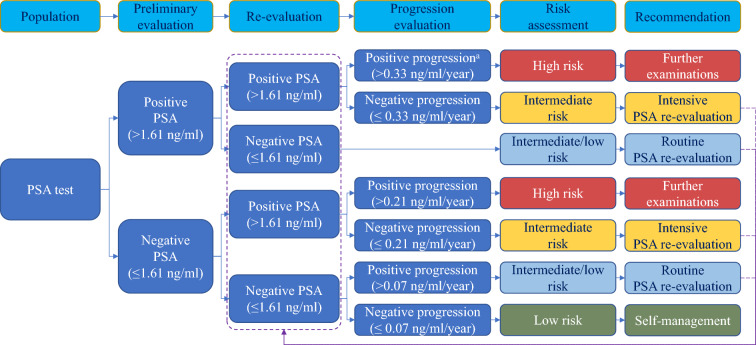
The recommendations flowchart of PSA screening for prostate cancer. a, absolute PSA velocity

Based on above screening performances, we recommended the optimized strategy three as the optimal screening strategy integrating population-based screening reference value and subgroup-specific progression indicator. As shown in Fig. 4, after at least two rounds of PSA test, participants were reclassified into subgroups with FR(−)/LR(−), FR(+)/LR(−), FR(−)/LR(+) and FR(+)/LR(+). After further progression evaluation, only participants with positive progression within either FR(−)/LR(+) or FR(+)/LR(+) was recommended to receive further examinations. Negative progression within either FR(−)/LR(+) or FR(+)/LR(+) were suggested to receive intensive re-evaluation of PSA, while regressive PSA and positive progression within FR(−)/LR(−) were recommended to receive routine re-evaluation of PSA. The left negative progression within FR(−)/LR(−) was recommended self-management.

## Discussion

To our knowledge, this is the first study to explore the PSA reference level for population-based PCa screening and the first study to investigate the optimal subgroup-specific PSA progression indicators and their corresponding subgroup-specific cut-off values under the setting of screening. Compared to traditional PSA screening, optimized risk-stratified multi-round PSA screening strategy integrating population-based PSA screening reference level and subgroup-specific progression indicators could not only reduce 32.4% of missed PCa, but also detected additional 8.0% of high-grade PCa than traditional screening strategy. Therefore, this optimized PSA screening strategy for PCa could be recommended as a fundamental strategy to improve the effectiveness of PCa screening.

In the PLCO trial, nearly 78.4% of PCa cases were missed under the traditional screening strategy (Table 2). Consistent with this study, several other trials adopting diagnostic criteria in population-based screening have also observed a large proportion of missed PCa [11, 27–29], including the Prostate Cancer Prevention Trial (PCPT), which missed 62.9% of PCa [30]. Since the average PSA level in healthy people is significantly lower than the diagnostic criteria of PSA for PCa patients, we proposed and calculated the population-based PSA screening reference value (1.61 ng/ml), which is similar to cut-off values (2.0 ng/ml) reported by Moul et al. [31]. Based on our proposed PSA screening reference value, we could avoid a large proportion of potential missed PCa. Meanwhile, when the participants were stratified according to the reference value, the incidence and mortality of PCa in the high-risk group were significantly higher than those in the corresponding low-risk group. These results preliminarily confirmed the relative rationality of the reference value. Moreover, when multi-round PSA tests were available, the participants can be further reclassified into four subgroups with FR(−)/LR(−), FR(+)/LR(−), FR(−)/LR(+) and FR(+)/LR(+), and the risk of PCa was significantly higher for any subgroups with positive PSA [including FR(+)/LR(−), FR(−)/LR(+) and FR(+)/LR(+)] compared with those with FR(−)/LR(−). This result also supported the clinical significance of multi-round PSA screening for PCa.

Since the PSA screening reference value is lower than the diagnostic criteria of PCa, the use of this reference value will inevitably lead to more false positives. To reduce unnecessary false positives and resulting biopsies, we further proposed the use of PSA progression indicators to identify participants with relatively higher risk of PCa from those with PSA above the screening reference value. Unlike previous studies focused on one progression indicator (such as PSA velocity or PSA doubling time) for the whole participants [32, 33], this study not only targeted different subgroups with elevated PSA and investigated subgroup-specific PSA progression indicators, but also compared the performances of different PSA progression indicators. Focusing on whole participants rather than specific subgroup would probably ignore the differences across different subgroups. In this study, we found that subgroup with FR(−)/LR(+) had significantly higher PCa incidence than subgroup with FR(+)/LR(−). More interestingly, we found that the subgroup with FR(−)/LR(+) had even higher PCa mortality risk than subgroup with FR(+)/LR(+). Similar subgroups were also proposed by Connolly et al. [34]. However, Connolly only focused on subgroups with PSA greater than or equal to 4.0 ng/ml and ignored those with PSA less than 4.0 ng/ml. Therefore, it is difficult for previous studies to find that subgroup with FR(−)/LR(+) has the highest PCa mortality than other comparable subgroups. This would be one of the important findings of this study. Further studies are needed to support this result in the future.

Furthermore, due to the inter-individual differences in baseline PSA and in screening interval between FR and LR PSA tests, as well as the intra-individual fluctuations between FR PSA and maximum PSA, we had investigated and compared the prediction accuracy of eight PSA progression indicators in selected subgroups with elevated PSA. Unexpectedly, we consistently found that absolute velocity rather than other indicators was the shared optimal progression indicator among the three subgroups with elevated PSA, while we did not find that the relative progression indicators or the maximum progression indicators were better than the absolute velocity. These results suggested that the impact of absolute velocity on long-time PCa incidence would overweigh maximum PSA, absolute PSA increment, and baseline PSA within the specific subgroup. These results further supported the necessity of investigating the subgroup-specific progression indicator and the importance of PSA progression velocity.

Due to the poor specificity of PSA screening for PCa [35], several other strategies were available for the risk management of patients with PSA abnormal levels and proposed to improve the detection rate of high-grade PCa, including Prostate Health Index (PHI) integrating total PSA, free PSA and [− 2] proPSA, volume-adjusted PSA combined with ultrasound, targeted biopsy with MRI and elevated other plasma protein (such as human kallikrein 2, β-microseminoprotein, and growth-differentiation factor-15), a polygenic risk score to identify population at high genetic risk of PCa, and their combination through artificial intelligence (AI) [15, 16, 36–43]. However, these methods require additional tests and technicians, which would inevitably increase the cost of screening and limited the promotion of these methods in regions with large populations and limited resources. To improve PSA screening performances without additional manpower and resources, we proposed three optimized screening strategies integrating population-based screening reference value and subgroup-specific progression indicators (Table 2). All three optimized screening strategies significantly improved the sensitivity and the detection rate of high-grade PCa compared to the traditional screening strategy. However, they also reduced the specificity and increase the number of false-positive cases. By pairwise comparison, the optimized strategy three could not only reduce missed diagnosis compared to the traditional PSA screening, but also could detect more high-grade PCa and reduce lots of false positive compared to other two optimized screening strategies. Therefore, we recommended the optimized strategy three as the optimal screening strategy and proposed the risk-stratified multi-round PSA screening flowchart based on this screening strategy (Fig. 4). Notably, we do not recommend diagnostic evaluation or intensive PSA re-evaluation for subgroup with positive progression within FR(−)/LR(−), since this subgroup represents a relatively large proportion of the population and has a non-significantly increased risk of PCa mortality. Alternatively, we recommend routine PSA re-evaluation for this subgroup to avoid unnecessary biopsies.

In addition to the important findings mentioned above, there are some limitations deserved attention. First, no independent external population are available to validate the findings observed in this study. These may limit the generalization of these results to other populations. However, the results of bootstrap resampling analyses with 2000 iterations well confirm the stability of all cut-off values used in the study. Second, rounds of screening between FR and LR PSA tests would have an impact on PSA progression. However, both multivariable analyses after adjusting rounds of screening and further interaction analyses between rounds of PSA screening and PSA status change found similar associations between PSA status change and PCa incidence as primary analyses. These analyses further suggested the stability of primary associations. Third, the interval between the FR and LR PSA test was not exactly the same for all participants in this study. Randazzo proposed that the screening interval should be 8 years when PSA < 1 ng/ml, and 4 years when PSA 1–2 ng/ml [44]. Overall, 89.9% of included participants in this study participated received at least four rounds of PSA tests with interval of at least four years. Moreover, the recommended cut-off value of PSA in this study was 1.61 ng/ml, which was in line with the screening recommendation of Randazzo in the interval of 4 years. In addition, the findings in this study would be very close to a real-world situation due to inclusion of participants with different screening intervals. Fifth, lowering the PSA cut-off level in population-based screening may lead to more false positives and overdiagnosis. To reduce the potential risk, we not only investigate subgroup-specific progression indicators, but also excluded definite regressive PSA and any subgroup-specific negative progressions as positive screen in recommended PSA screening flowchart. As observed, the optimized screening strategy not only reduced the missed PCa, but also improved the detection of high-grade PCa and reduced lots of false positive. Finally, several factors could potentially limit the choice of cut-offs of PSA and its progression indictors, including the PSA tests methods, population heterogeneity, and analytical variability. For the different PSA test methods, such as Tandem R, Tandem E, and IMx PSA, previous researches showed no significant differences between different methods [45, 46]. Therefore, their potential impact on the cut-off value could be ignored. However, the heterogeneity of the population, including cross-racial and intra-racial heterogeneity, could indeed affect the choice of cut-off values between different population and subgroups by ages and races [47, 48]. Analytical variability could also lead to different cut-off values between different subgroups within the same population, especially in the context of small sample size. Therefore, further more studies with sophisticated design and large sample size are needed to validate the current results in the future.

In conclusion, in this study, we have proposed a risk-stratified multi-round PSA screening strategy integrating population-based screening reference level and subgroup-specific progression indicator. This optimized screening strategy will not only reduce potential missed diagnosis of PCa, but also improve the detection of high-grade PCa and reduced false positive. More importantly, it requires no additional costs. Therefore, it could be recommended as a fundamental strategy to improve screening effectiveness for PCa in regions or countries with high PCa burden but limited resources. In the future, interdisciplinary interactions in clinical practice deserve more attention to improve the detection of high-risk PCa, reduce unnecessary biopsies, and ultimately improve the quality of life of patients. If available and feasible, other examinations or tests (such as multiparametric MRI or liquid biopsy [49]) in combination with the optimized PSA screening strategy, or their combination through AI should be recommended to further reduce false positives and potential overdiagnosis.

## Supplementary Information

**Additional file 1: Figure S1.** Flowchart of participants’ selection. **Figure S2.** Kaplan–Meier curves of cumulative prostate cancer incidence (A) and mortality (B) with first-round PSA for subgroups based on population-based screening reference value. **Figure S3.** Kaplan–Meier curves of cumulative prostate cancer incidence (A, B, C) and mortality (D, E, F) for subgroups with FR(−)/LR(−), FR(−)/LR(+) and FR(+)/LR(+) stratified by subgroup-specific PSA progression indicator. **Figure S4.** Cumulative incidence (A) and mortality (B) of prostate cancer (PCa) between different subgroups integrating the screening reference level and subgroup-specific progression indicators. **Table S1.** Baseline characteristics associated with the incidence of prostate cancer. **Table S2.** Bootstrap resampling analyses on PSA screening reference level and the cut-off values of subgroup-specific progression indicators with 2000 iterations. **Table S3.** Association of PSA status change with prostate cancer (PCa) incidence and mortality. **Table S4.** Interaction between rounds of PSA screening and PSA status change on prostate cancer (PCa) incidence and mortality. **Table S5.** Association of PSA status change integrated progression indicator with prostate cancer (PCa) incidence and mortality.

## Data Availability

The data are available on application to the PLCO trial (https://cdas.cancer.gov/plco/).
