# Supplementary material for: Risk-stratified multi-round PSA screening for prostate cancer integrating the screening reference level and subgroup-specific progression indicators
Source: Eur J Med Res. 2023 Jul 26;28:257. doi: 10.1186/s40001-023-01228-x (PMC10369696; doi:10.1186/s40001-023-01228-x)
Supplement: Supplementary file 1 — Additional file 1: Figure S1. Flowchart of participants’ selection. Figure S2. Kaplan–Meier curves of cumulative prostate cancer incidence (A) and mortality (B) with first-round PSA for subgroups based on population-based screening reference value. Figure S3. Kaplan–Meier curves of cumulative prostate cancer incidence (A, B, C) and mortality (D, E, F) for subgroups with FR(−)/LR(−), FR(−)/LR(+) and FR(+)/LR(+) stratified by subgroup-specific PSA progression indicator. Figure S4. Cumulative incidence (A) and mortality (B) of prostate cancer (PCa) between different subgroups integrating the screening reference level and subgroup-specific progression indicators. Table S1. Baseline characteristics associated with the incidence of prostate cancer. Table S2. Bootstrap resampling analyses on PSA screening reference level and the cut-off values of subgroup-specific progression indicators with 2000 iterations. Table S3. Association of PSA status change with prostate cancer (PCa) incidence and mortality. Table S4. Interaction between rounds of PSA screening and PSA status change on prostate cancer (PCa) incidence and mortality. Table S5. Association of PSA status change integrated progression indicator with prostate cancer (PCa) incidence and mortality. [file 40001_2023_1228_MOESM1_ESM.docx]

**Figure S1.** Flowchart of participants’ selection.

**Figure S2.** Kaplan-Meier curves of cumulative prostate cancer incidence (A) and mortality (B) with first-round PSA for subgroups based on population-based screening reference value.

**Figure S3.** Kaplan-Meier curves of cumulative prostate cancer incidence (A, B, C) and mortality (D, E, F) for subgroups with FR(-)/LR(-), FR(-)/LR(+) and FR(+)/LR(+) stratified by subgroup-specific PSA progression indicator.

**Figure S4.** Cumulative incidence (A) and mortality (B) of prostate cancer (PCa) between different subgroups integrating the screening reference level and subgroup-specific progression indicators.

**Table S1.** Baseline characteristics associated with the incidence of prostate cancer.

**Table S2.** Bootstrap resampling analyses on PSA screening reference level and the cut-off values of subgroup-specific progression indicators with 2000 iterations.

**Table S3.** Association of PSA status change with prostate cancer (PCa) incidence and mortality.

**Table S4.** Interaction between rounds of PSA screening and PSA status change on prostate cancer (PCa) incidence and mortality.

**Table S5.** Association of PSA status change integrated progression indicator with prostate cancer (PCa) incidence and mortality.

**Figure S1. Flowchart of participants’ selection.**


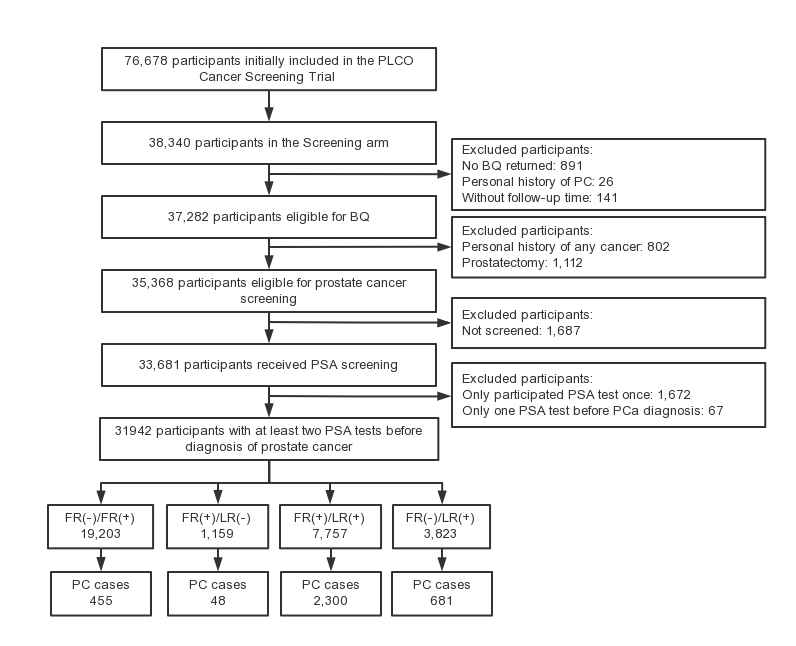


**Figure S2. Kaplan-Meier curves of cumulative prostate cancer incidence (A) and mortality (B) with first-round PSA for subgroups based on population-based screening reference value.**

**
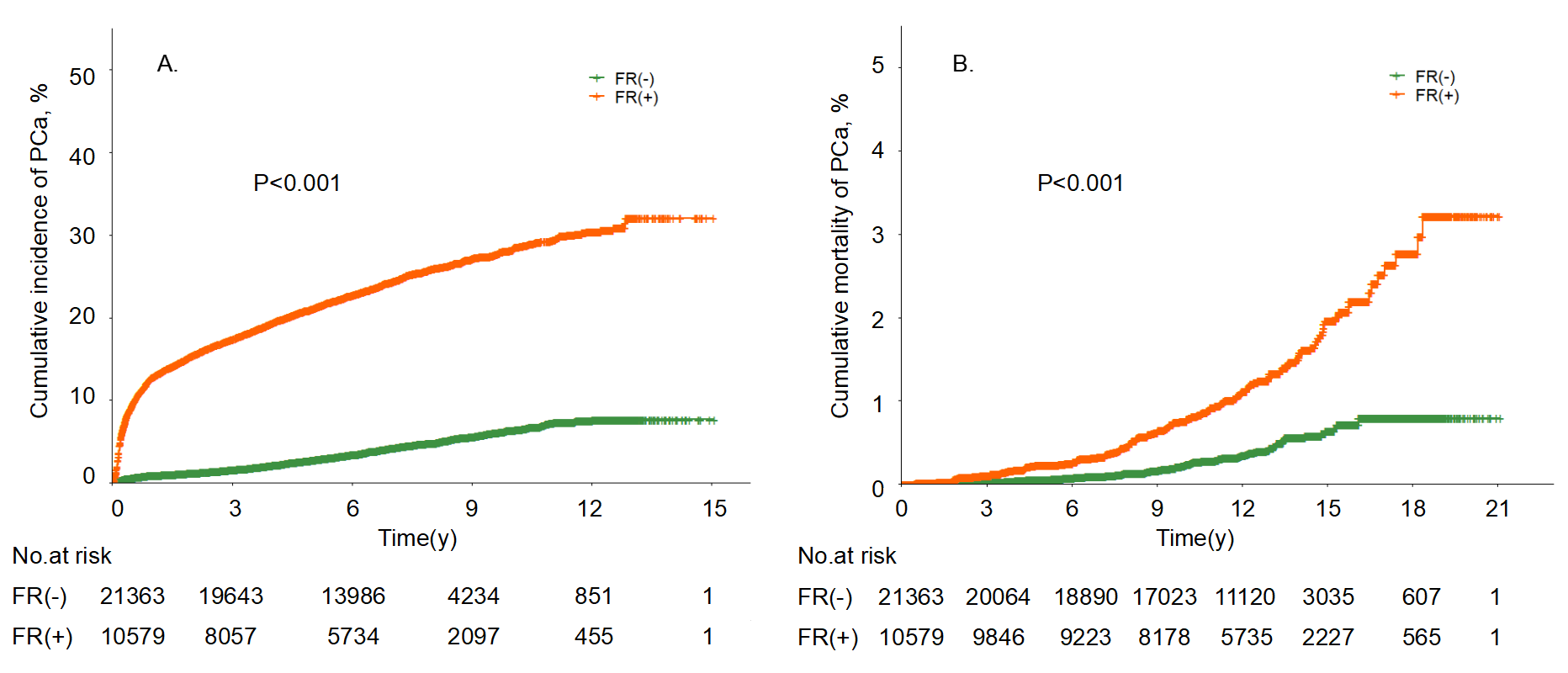
**

Abbreviations: FR(+), first-round positive PSA; FR(-), first-round negative PSA.

**Figure S3. Kaplan-Meier curves of cumulative prostate cancer incidence (A, B, C) and mortality (D, E, F) for subgroups with FR(-)/LR(-), FR(-)/LR(+) and FR(+)/LR(+) stratified by subgroup-specific PSA progression indicator.**

**
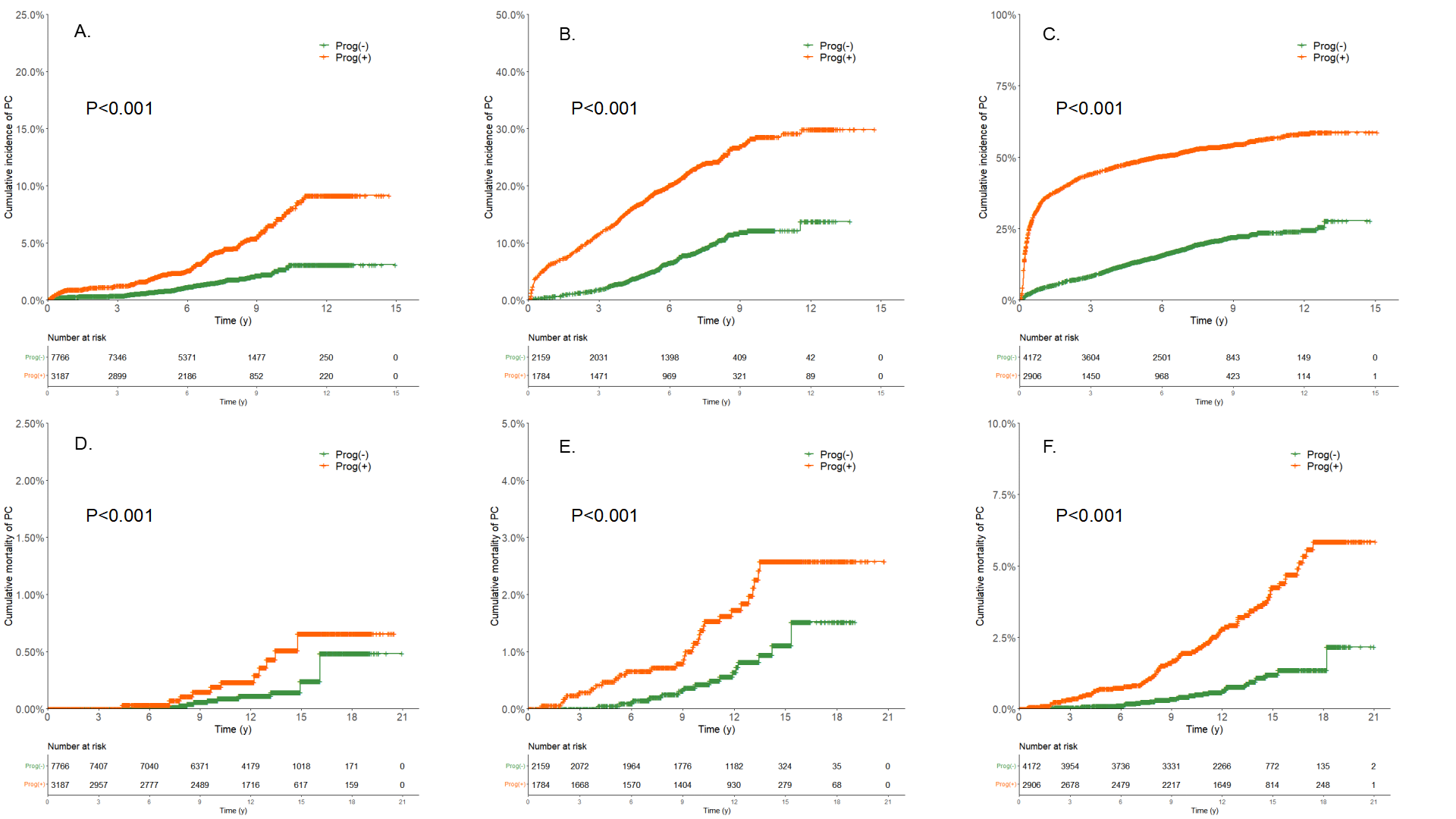
**

Abbreviations: FR(-)/LR(-), stable negative PSA; FR(-)/LR(+), gain of positive; FR(+)/LR(+) stable positive PSA; Prog(+) and Prog(-), PSA increment greater than and less than (including equal to) subgroup-specific cut-off values of optimal progression index.

**Figure S4. Cumulative incidence (A) and mortality (B) of prostate cancer (PCa) between different subgroups integrating the screening reference level and subgroup-specific progression indicators.**


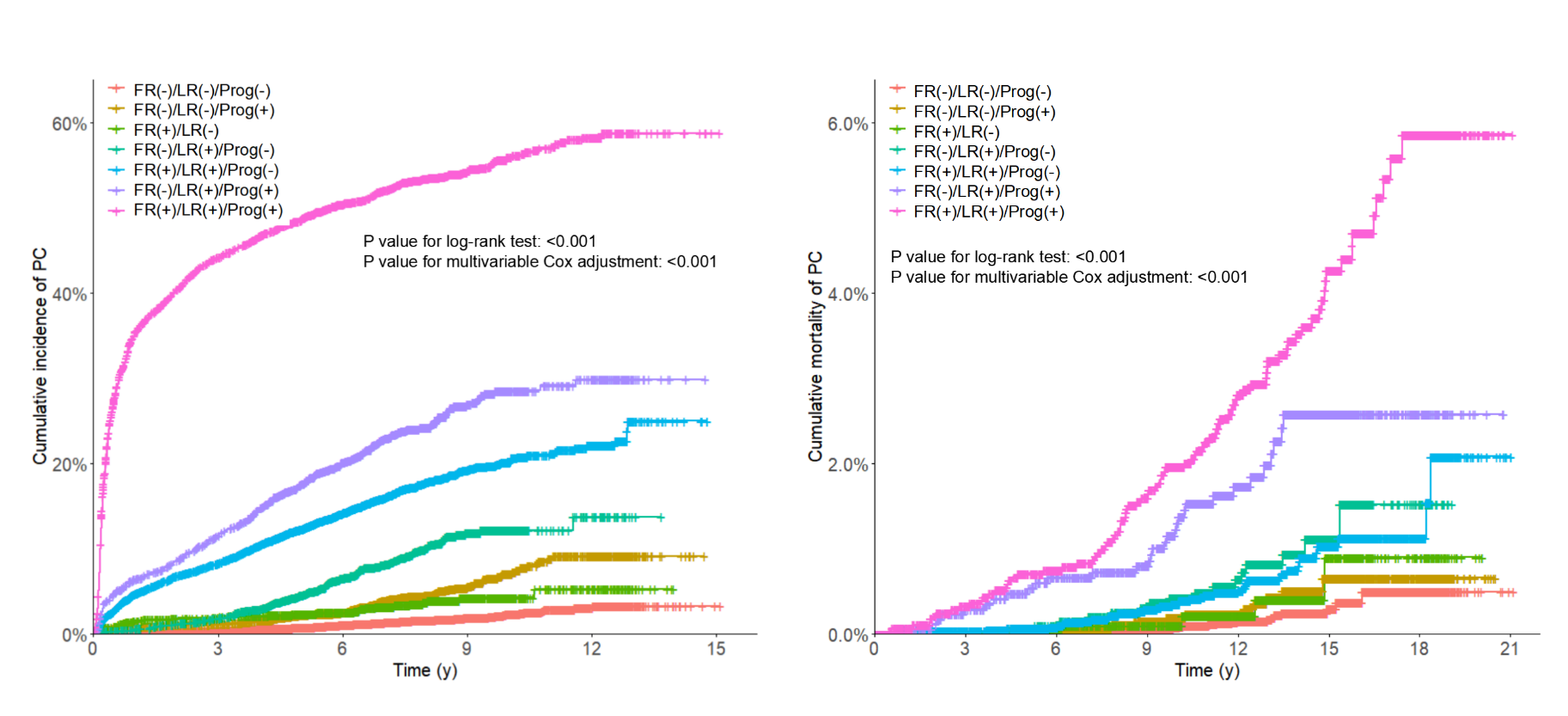


Note: FR(-)/LR(-), stable negative PSA; FR(+)/LR(-), loss of positive PSA; FR(-)/LR(+), gain of positive; FR(+)/LR(+) stable positive PSA; Prog (+)/(-): positive/negative progression. *, adjusted all potentially confounding factors mentioned in the method.

**Table S1. Baseline characteristics associated with the incidence of prostate cancer.**

| Subgroups^a^ | Participants,  No. (%) | PC cases,  No. (%) | Follow-up,  1000 PYs | IR of PC,  per 1000 PYs | P value for  K-M curve | Adjusted  HR (95%CI) ^b^ | P value ^b^ |
| --- | --- | --- | --- | --- | --- | --- | --- |
| Age at entrance | | | | | |  |  |
| <60 years | 10741 (33.6) | 869(24.9) | 66.44 | 13.08 | <0.001 | Ref. |  |
| 60-70 years | 17442 (54.6) | 2184(62.7) | 121.01 | 18.05 |  | 1.11(1.02-1.21) | 0.016 |
| ≥ 70 years | 3759 (11.8) | 431(12.4) | 23.52 | 18.33 |  | 0.96(0.85-1.09) | 0.549 |
| Race | | | | | |  |  |
| White | 28306 (88.6) | 3124(89.7) | 187.58 | 16.65 | <0.001 | Ref. |  |
| Black | 1368 (4.3) | 183(5.3) | 7.65 | 23.93 |  | 1.35(1.16-1.57) | <0.001 |
| Other | 2268 (7.0) | 176(5.0) | 15.65 | 11.25 |  | 0.77(0.66-0.90) | 0.001 |
| Body mass index | | | | | |  |  |
| 0-25 kg/m^2^ | 8370 (26.2) | 964(27.7) | 56.35 | 17.11 | 0.003 | Ref. |  |
| 25-30 kg/m^2^ | 15925 (49.9) | 1781(51.1) | 106.06 | 16.79 |  | 1.03(0.95-1.11) | 0.506 |
| >30 kg/m^2^ | 7297 (22.8) | 694(19.9) | 46.25 | 15.01 |  | 0.96(0.86-1.06) | 0.361 |
| Smoking state | | | | | |  |  |
| Never | 11937 (37.4) | 1485(42.6) | 80.79 | 18.38 | <0.001 | Ref. |  |
| Current | 3522 (11.0) | 298(8.6) | 20.91 | 14.25 |  | 0.64(0.56-0.73) | <0.001 |
| Former | 16476 (51.6) | 1699(48.8) | 109.21 | 15.56 |  | 0.81(0.76-0.87) | <0.001 |
| Family history of prostate cancer | | | | | |  |  |
| No | 28811 (90.2) | 3029(86.9) | 190.82 | 15.87 | <0.001 | Ref. |  |
| Yes | 2361 (7.4) | 373(10.7) | 15.19 | 24.56 |  | 1.51(1.36-1.68) | <0.001 |
| History of previous PSA screening | | | | | |  |  |
| No | 14810 (46.4) | 1449(41.6) | 95.28 | 15.21 | <0.001 | Ref. |  |
| 1 time | 11449 (35.8) | 1302(37.4) | 75.30 | 17.29 |  | 1.11(1.03-1.20) | 0.006 |
| ≥ 2 times | 2881 (9.0) | 451(12.9) | 22.19 | 20.32 |  | 1.03(0.92-1.16) | 0.576 |
| Enlarged prostate | | | | | |  |  |
| No | 25619 (80.2) | 2648(76.0) | 168.66 | 15.70 | <0.001 | Ref. |  |
| Yes | 6301 (19.7) | 833(23.9) | 42.13 | 19.77 |  | 1.03(0.95-1.12) | 0.511 |
| History of diabetes | | | | | |  |  |
| No | 29114 (91.1) | 3277(94.1) | 193.63 | 16.92 | <0.001 | Ref. |  |
| Yes | 2731 (8.5) | 196(5.6) | 16.60 | 11.81 |  | 0.62(0.54-0.72) | <0.001 |
| FR DRE |  |  |  |  |  |  |  |
| Negative | 16376 (50.3) | 1552(44.5) | 108.75 | 14.27 | <0.001 | Ref. |  |
| Positive | 15309 (47.0) | 1839(52.8) | 96.71 | 19.02 |  | 1.15(1.07-1.24) | <0.001 |
| LR DRE |  |  |  |  |  |  |  |
| Negative | 12375(38.7) | 1032(29.6) | 82.96 | 12.44 | <0.001 | Ref. |  |
| Positive | 18689(58.5) | 2359(67.7) | 122.50 | 19.26 |  | 1.47(1.36-1.60) | <0.001 |
| Rounds of PSA screening | |  |  |  |  |  |  |
| 2 rounds | 1508(4.7) | 451(12.9) | 7.14 | 63.20 | <0.001 | Ref. |  |
| 3 rounds | 1729(5.4) | 457(13.1) | 9.14 | 49.98 |  | 0.72(0.63-0.82) | <0.001 |
| 4 rounds | 4147(13.0) | 726(20.8) | 31.93 | 22.74 |  | 0.33(0.30-0.38) | <0.001 |
| 5 rounds | 6420(20.1) | 674(19.3) | 9.14 | 73.71 |  | 0.19(0.17-0.21) | <0.001 |
| 6 rounds | 18138(56.8) | 1176(33.8) | 116.02 | 10.14 |  | 0.13(0.11-0.14) | <0.001 |

Abbreviations: PC, prostate cancer; PY, person-year; IR, incidence rate; FR DRE, first-round digital rectal examination; LR DRE, last-round digital examination.

^a^Missing data in the index variable were not shown.

^b^adjusted all index variables listed in the table.

**Table S2. Bootstrap resampling analyses on PSA screening reference level and the cut-off values of subgroup-specific progression indicators with 2000 iterations.**

| Cut-off value | Participants | Events | Optimal value | 95%CI |
| --- | --- | --- | --- | --- |
| PSA screening reference level | 21279/10663 | 911/2573 | 1.60 | 1.47-1.86 |
| Subgroup-specific cut-off value of absolute velocity | | | | |
| Subgroup with FR(-)/LR(-) | 7766/3187 | 121/142 | 0.07 | 0.06-0.10 |
| Subgroup with FR(-)/LR(+) | 2375/1568 | 217/360 | 0.23 | 0.20-0.28 |
| Subgroup with FR(+)/LR(+) | 4444/2634 | 856/1389 | 0.37 | 0.31-0.42 |

Abbreviations: 95%CI, 95% confidential interval; FR(-)/LR(-), stable negative PSA; FR(-)/LR(+), gain of positive; FR(+)/LR(+) stable positive PSA.

**Table S3. Association of PSA status change with prostate cancer (PCa) incidence and mortality.**

| Subgroups | Participants,  No. (%) | Event,  No. (%) | Follow-up,  1000 PYs | Event rate,  per 1000 PYs | Unadjusted  HR (95%CI) | P value for  log-rank test | Adjusted  HR (95%CI) ^a^ | P value ^a^ |
| --- | --- | --- | --- | --- | --- | --- | --- | --- |
| PCa incidence | 31942(100.0) | 3484(100.0) | 210.96 | 16.51 |  |  |  |  |
| FR(-)/LR(-) | 17420(54.5) | 343(9.8) | 121.99 | 2.81 | Ref. | <0.001 | Ref. |  |
| FR(+)/LR(-) | 1257(3.9) | 41(1.2) | 8.56 | 4.79 | 1.69(1.23-2.34) |  | 1.66(1.20-2.29) | 0.002 |
| FR(-)/LR(+) | 3943(12.3) | 577(16.6) | 25.96 | 22.22 | 7.78(6.81-8.89) |  | 8.29(7.25-9.48) | <0.001 |
| FR(+)/LR(+) | 9322(29.2) | 2523(72.4) | 54.45 | 46.34 | 15.90(14.20-17.80) |  | 14.52(12.95-16.28) | <0.001 |
| PCa mortality | 31942(100.0) | 216(100.0) | 367.29 | 0.59 |  |  |  |  |
| FR(-)/LR(-) | 17420(54.5) | 35(16.2) | 198.61 | 0.18 | Ref. | <0.001 | Ref. |  |
| FR(+)/LR(-) | 1257(3.9) | 4(1.9) | 13.65 | 0.29 | 1.52(0.54-4.29) |  | 1.47(0.52-4.15) | 0.465 |
| FR(-)/LR(+) | 3943(12.3) | 47(21.8) | 45.75 | 1.03 | 5.60(3.61-8.68) |  | 5.71(3.68-8.86) | <0.001 |
| FR(+)/LR(+) | 9322(29.2) | 130(60.2) | 109.24 | 1.19 | 5.48(3.74-8.03) |  | 5.01(3.41-7.37) | <0.001 |

Abbreviations: PY, person-year; HR (95%CI), hazard ratio (95% confidential interval); FR(-)/LR(-), stable negative PSA; FR(+)/LR(-), loss of positive PSA; FR(-)/LR(+), gain of positive; FR(+)/LR(+) stable positive PSA.

^a^adjusted all potentially confounding factors mentioned in the method.

**Table S4.** **Interaction between rounds of PSA screening and PSA status change on prostate cancer (PCa) incidence and mortality.**

| Rounds of PSA screening | PSA status | Participants,  No. (%) | PCa cases,  No. (%) | Adjusted  HR (95%CI)^a^ | P value ^a^ | PCa deaths,  No. (%) | Adjusted  HR (95%CI)^a^ | P value ^a^ |
| --- | --- | --- | --- | --- | --- | --- | --- | --- |
| 2 rounds | FR(-)/LR(-) | 651(43.2) | 25(5.5) | Ref. |  | 1(4.2) | Ref. |  |
|  | FR(+)/LR(-) | 68(4.5) | 10(2.2) | 4.91(2.35-10.25) | <0.001 | 0(0.0) | - | - |
|  | FR(-)/LR(+) | 87(5.8) | 19(4.2) | 7.09(3.89-12.92) | <0.001 | 0(0.0) | - | - |
|  | FR(+)/LR(+) | 702(46.6) | 397(88.0) | 19.09(12.68-28.76) | <0.001 | 23(95.8) | 16.58(2.17-126.86) | 0.007 |
| 3 rounds | FR(-)/LR(-) | 779(45.1) | 37(8.1) | Ref. |  | 2(9.5) | Ref. |  |
|  | FR(+)/LR(-) | 69(4.0) | 4(0.9) | 1.21(0.43-3.41) | 0.716 | 0(0.0) | - | - |
|  | FR(-)/LR(+) | 151(8.7) | 41(9.0) | 6.02(3.85-9.40) | <0.001 | 2(9.5) | 4.60(0.64-33.32) | 0.131 |
|  | FR(+)/LR(+) | 730(42.2) | 375(82.1) | 13.70(9.72-19.30) | <0.001 | 17(81.0) | 6.34(1.43-28.17) | 0.015 |
| 4 rounds | FR(-)/LR(-) | 2019(48.7) | 85(11.7) | Ref. |  | 11(19.3) | Ref. |  |
|  | FR(+)/LR(-) | 169(4.1) | 11(1.5) | 1.65(0.88-3.10) | 0.119 | 1(1.8) | 1.10(0.14-8.57) | 0.931 |
|  | FR(-)/LR(+) | 407(9.8) | 74(10.2) | 4.63(3.39-6.33) | <0.001 | 8(14.0) | 3.37(1.35-8.42) | 0.009 |
|  | FR(+)/LR(+) | 1552(37.4) | 556(76.6) | 10.83(8.59-13.66) | <0.001 | 37(64.9) | 4.36(2.19-8.68) | <0.001 |
| 5 rounds | FR(-)/LR(-) | 3291(51.3) | 59(8.8) | Ref. |  | 7(15.6) | Ref. |  |
|  | FR(+)/LR(-) | 278(4.3) | 8(1.2) | 1.71(0.82-3.58) | 0.155 | 1(2.2) | 1.64(0.20-13.38) | 0.645 |
|  | FR(-)/LR(+) | 827(12.9) | 129(19.1) | 8.97(6.58-12.22) | <0.001 | 12(26.7) | 6.90(2.70-17.62) | <0.001 |
|  | FR(+)/LR(+) | 2024(31.5) | 478(70.9) | 15.89(12.08-20.91) | <0.001 | 25(55.6) | 4.83(2.05-11.37) | <0.001 |
| 6 rounds | FR(-)/LR(-) | 10680(58.9) | 137(11.6) | Ref. |  | 14(20.3) | Ref. |  |
|  | FR(+)/LR(-) | 673(3.7) | 8(0.7) | 0.96(0.47-1.96) | 0.911 | 2(2.9) | 2.22(0.50-9.78) | 0.294 |
|  | FR(-)/LR(+) | 2471(13.6) | 314(26.7) | 10.37(8.48-12.68) | <0.001 | 25(36.2) | 7.20(3.73-13.89) | <0.001 |
|  | FR(+)/LR(+) | 4314(23.8) | 717(61.0) | 14.31(11.90-17.24) | <0.001 | 28(40.6) | 4.13(2.15-7.94) | <0.001 |

Abbreviations: HR (95%CI), hazard ratio (95% confidential interval); FR(-)/LR(-), stable negative PSA; FR(+)/LR(-), loss of positive PSA; FR(-)/LR(+), gain of positive; FR(+)/LR(+) stable positive PSA.

^a^adjusted all potentially confounding factors mentioned in the method.

**Table S5. Association of PSA status change integrated progression indicator with prostate cancer (PCa) incidence and mortality.**

| Subgroups | Participants,  No. (%) | Event,  No. (%) | Follow-up,  1000 PYs | Event rate,  per 1000 PYs | Adjusted  HR (95%CI)^a^ | P value^a^ |
| --- | --- | --- | --- | --- | --- | --- |
| PCa incidence | 31942(100.0) | 3484(100.0) | 210.96 | 16.51 |  |  |
| FR(-)/LR(-)/Prog(-) | 14233(44.6) | 201(5.8) | 99.02 | 2.03 | Ref. |  |
| FR(-)/LR(-)/Prog(+) | 3187(10.0) | 142(4.1) | 22.97 | 6.18 | 2.68(2.16-3.33) | <0.001 |
| FR(+)/LR(-) | 1257(3.9) | 41(1.2) | 8.56 | 4.79 | 2.29(1.64-3.21) | <0.001 |
| FR(-)/LR(+)/Prog(-) | 2159(6.8) | 181(5.2) | 22.97 | 7.88 | 6.41(5.24-7.84) | <0.001 |
| FR(+)/LR(+)/Prog(-) | 6416(20.1) | 1038(29.8) | 42.42 | 24.47 | 11.61(9.97-13.52) | <0.001 |
| FR(-)/LR(+)/Prog(+) | 1784(5.6) | 396(11.4) | 11.08 | 35.75 | 16.34(13.78-19.37) | <0.001 |
| FR(+)/LR(+)/Prog(+) | 2906(9.1) | 1485(42.6) | 12.03 | 123.42 | 44.53(38.30-51.77) | <0.001 |
| PCa mortality | 31942(100.0) | 216(100.0) | 367.29 | 0.59 |  |  |
| FR(-)/LR(-)/Prog(-) | 14233(44.6) | 24(11.1) | 161.78 | 0.15 | Ref. |  |
| FR(-)/LR(-)/Prog(+) | 3187(10.0) | 11(5.1) | 36.83 | 0.30 | 1.78(0.87-3.64) | 0.116 |
| FR(+)/LR(-) | 1257(3.9) | 4(1.9) | 13.65 | 0.29 | 1.74(0.60-5.04) | 0.304 |
| FR(-)/LR(+)/Prog(-) | 2159(6.8) | 16(7.4) | 36.83 | 0.43 | 4.27(2.27-8.05) | <0.001 |
| FR(+)/LR(+)/Prog(-) | 6416(20.1) | 41(19.0) | 74.72 | 0.55 | 3.02(1.82-5.04) | <0.001 |
| FR(-)/LR(+)/Prog(+) | 1784(5.6) | 31(14.4) | 20.45 | 1.52 | 9.14(5.35-15.61) | <0.001 |
| FR(+)/LR(+)/Prog(+) | 2906(9.1) | 89(41.2) | 34.52 | 2.58 | 12.15(7.56-19.51) | <0.001 |

Abbreviations: HR (95%CI), hazard ratio (95% confidential interval); FR(-)/LR(-), stable negative PSA; FR(+)/LR(-), loss of positive PSA; FR(-)/LR(+), gain of positive; FR(+)/LR(+), stable positive PSA; Prog, progression; -, negative; +, positive.

^a^adjusted all potentially confounding factors mentioned in the method.
